# Fatal Form of COVID-19 in a Young Male Bodybuilder Anabolic Steroid Using: The First Autopsied Case

**DOI:** 10.3390/medicina58101373

**Published:** 2022-09-29

**Authors:** Costel Vasile Siserman, Ionuț Isaia Jeican, Dan Gheban, Vlad Anton, Daniela Mironescu, Sergiu Șușman, Mihaela Laura Vică, Mihaela Lazăr, Maria Aluaș, Corneliu Toader, Silviu Albu

**Affiliations:** 1 Institute of Legal Medicine, 400006 Cluj-Napoca, Romania; 2 Department of Legal Medicine, Iuliu Hatieganu University of Medicine and Pharmacy, 400006 Cluj-Napoca, Romania; 3 Department of Anatomy and Embryology, Iuliu Hatieganu University of Medicine and Pharmacy, 400006 Cluj-Napoca, Romania; 4 Infectious Disease Clinical Hospital, 400000 Cluj-Napoca, Romania; 5 Department of Pathology, Iuliu Hatieganu University of Medicine and Pharmacy, 400006 Cluj-Napoca, Romania; 6 Department of Pathology, Emergency Clinical Hospital for Children, 400370 Cluj-Napoca, Romania; 7 Department of Medical Biochemistry, Iuliu Hatieganu University of Medicine and Pharmacy, 400349 Cluj-Napoca, Romania; 8 Imogen Medical Research Institute, County Clinical Emergency Hospital, 400000 Cluj-Napoca, Romania; 9 Department of Histology, Iuliu Hatieganu University of Medicine and Pharmacy, 400349 Cluj-Napoca, Romania; 10 Department of Cell and Molecular Biology, Iuliu Hatieganu University of Medicine and Pharmacy, 400349 Cluj-Napoca, Romania; 11 Viral Respiratory Infections Laboratory, Cantacuzino National Military-Medical Institute for Research and Development, 050096 Bucharest, Romania; 12 Department of Oral Health, Iuliu Hatieganu University of Medicine and Pharmacy, Victor Babeș Str., No. 15, 400012 Cluj-Napoca, Romania; 13 Clinic of Neurosurgery, National Institute of Neurology and Neurovascular Diseases, 041914 Bucharest, Romania; 14 Department of Head and Neck Surgery and Otorhinolaryngology, University Clinical Hospital of Railway Company, Iuliu Hatieganu University of Medicine and Pharmacy, 400015 Cluj-Napoca, Romania

**Keywords:** anabolic androgenic steroids, testosterone, COVID-19, risk factor, autopsy

## Abstract

We report the case of a 34-year-old male patient, a bodybuilding trainer and user of anabolic androgenic steroids (AASs) for 16 years. He was found in cardio-respiratory arrest in his home. By performing a medico-legal autopsy, a severe form of COVID-19, aortic atherosclerotic plaques, and an old myocardial infarction was found. The SARS-CoV-2 RT-PCR test on necroptic lung fragments was positive, with a B.1.258 genetic line. The histopathological examinations showed microthrombi with endothelitis in the cerebral tissue, massive pulmonary edema, diffuse alveolar damage grade 1, pulmonary thromboembolism, hepatic peliosis, and severe nesidioblastosis. The immunohistochemical examinations showed SARS-CoV-2 positive in the myocardium, lung, kidneys, and pancreas. ACE-2 receptor was positive in the same organs, but also in the spleen and liver. HLA alleles A*03, A*25, B*18, B*35, C*04, C*12, DRB1*04, DRB1*15, DQB1*03, DQB1*06 were also identified. In conclusion, death was due to a genetic predisposition, a long-term abuse of AASs that favored the development of a pluriorganic pathological tissue terrain, and recent consumption of AASs, which influenced the immune system at the time of infection.

## 1. Introduction

Anabolic androgenic steroids (AASs) are widely used illicitly to improve body appearance and increase strength [1], but they can lead to adverse side effects on immune function [2]. The link between COVID-19 and AASs has not been extensively documented to date. The only two references to the topic are results published in a cross-sectional study on 39 current AASs users (of 520 total study participants) [3] and in a case report on a severe COVID-19 male patient using AASs [4]. These data appear to be supported by the link between androgen signaling and SARS-CoV-2 infectivity, and point to AASs as a risk factor for severe COVID-19.

The aim of the study was to analyze the autopsy results of a young bodybuilder patient, who consumed AASs regularly and was affected by a severe form of COVID-19.

## 2. Materials and Methods

**Histopathology**. For the histopathological examination of the organ fragments collected at autopsy, the samples were fixed in formaldehyde 7% for 5 days, after which the samples were oriented and placed in cassettes. Tissue processing was performed using a vacuum infiltration processor, Tissue-Tek VIP 5 Jr (Sakura, Alphen aan den Rijn, The Netherlands). Paraffin embedding and sectioning were performed using the Tissue-Tek TEC 6 system (Sakura, Alphen aan den Rijn, The Netherlands) and Accu-Cut SRM 200 Rotary Microtome (Sakura, Alphen aan den Rijn, The Netherlands). Slide staining was performed using the automated slide stainer Tissue-Tek Prisma Plus (Sakura, Alphen aan den Rijn, The Netherlands) according to the internal staining protocol, using Mayer Modified Hematoxylin (Titolchimica, Rovigo, Italy) and Eosin solution (10 g Eosin B in 1000 mL distilled water).

Immunohistochemistry was performed automatically on 3-μm-thick sections of formalin-fixed and paraffin-embedded tissues with MD Stainer (Vitro Master Diagnostica^®^, Granada, Spain) using ethylenediaminetetraacetic acid (EDTA), at pH  =  9, for antigen retrieval. For the immunohistochemical assessment, we used Anti-SARS Coronavirus NP Mouse anti-Virus antibody (clone B46F, Invitrogen, Waltham, Massachusetts, USA) at a 1:100 dilution, and anti-ACE antibody (clone15348, Abcam, Cambridge, UK) at 1:100 dilution.

Microscopic examination was performed by an experienced pathologist (D.G.) using an Olympus BX46 clinical microscope (Olympus Europe SE & Co, Hamburg, Germany) with dedicated image acquisition camera and software. All sections were examined at 400× magnification.

**Molecular biology.** During autopsy, 3 lung fragments were collected to perform the SARS-CoV-2 RT-PCR test. Total nucleic acids isolation was performed with MasterPure™ Complete DNA and RNA Purification Kit (EPICENTRE Biotechnologies, Madison, WI, USA), according to the manufacturer’s instructions, using the protocol for total nucleic acids purification from tissue samples. A Pearl Nanophotometer (Implen GmbH, Munich, Germany) was engaged in determining the DNA and RNA concentration and purity. The RNA sample was amplified on a QuantStudio™ 5 RT-PCR System (Thermo Fisher Scientific Inc., Waltham, MA, USA) after reverse transcription, using aSARS-CoV-2 Real-TM kit (Sacace Biotechnologies, Como, Italy). This multiplex RT-PCR assay uses four simultaneous amplification reactions: those of the E gene region common for all SARS-like coronaviruses (FAM channel), the specific SARS-CoV-2 E gene (ROX channel) and the specific SARS-CoV-2 N gene (Cy5 channel), as well as the amplification of the nucleic acid sequence of the Internal Control-RNA (HEX channel). The assay also included a positive control of amplification (cDNA C+). The following program was used: reverse transcription for 20 min at 35 °C, initial denaturation for 5 min at 94 °C, 5 amplification cycles (10 s at 94 °C and 25 s at 64 °C) followed by 45 amplification cycles (10 s at 94 °C and 25 s at 64 °C with fluorescence detection).

Using the initial solution of isolated total nucleic acids, the identification of SARS-CoV-2 variant was performed. RNA preparation and amplification were carried out in accordance with protocols published by the ARTIC network, using the V3 version of the ARTIC primer set from Integrated DNA Technologies (Coralville, IA, USA) to create tiled amplicons across the SARS-CoV-2 genome. Libraries were prepared using the Nextera DNA Flex library preparation kit and MiSeq reagent cartridge V2 (Illumina, San Diego, CA, USA).

Using the same initial solution, the identification of HLA-A, B, C, DRB1 and DQB1 was performed. HLA-FluoGene ABC kit and HLA-FluoGene DRDQ kit (inno-train Diagnostik GmbH, Kronberg, Germany) were used according to the manufacturer’s instructions, based on the Sequence Specific Priming Polymerase Chain Reaction (SSP-PCR). DNA amplification was carried out on a G-Storm thermal cycler (Gene Technologies Ltd., Essex, UK) and the mixture containing the extracted DNA sample was submitted to 40 amplification cycles (15 s at 96 °C and 60 s at 60 °C) after an initial denaturation step for 2 min at 95 °C. Detection of the PCR products was performed by measuring fluorescence signals on a FluoVista Analyzer (inno-train Diagnostik GmbH, Kronberg, Germany), the endpoint fluorescence of the various fluorochromes before and after PCR was automatically calculated using the FluoGene analysis software.

## 3. Case Presentation

A 34-year-old patient, a fitness and bodybuilding trainer, was found by the ambulance in cardio-respiratory arrest in his home. The patient did not respond to resuscitation maneuvers. A medico-legal autopsy was requested to establish the causes of death.

From the **postmortem heteroanamnesis**, the following information was recorded:

- 4 days before death, the patient presented infectious symptomatology with sudden onset (altered general condition, fever 38 °C, curvature, nausea, vomiting, dry cough, dyspnea whose intensity increased progressively); about 15 min before death, the patient presented anxiety, obnubilation, severe dyspnea; death occurred suddenly;
- 3 days before the onset of the mentioned symptomatology, the patient encountered COVID-19-positive subjects in the gym room;
- during the 4 days of illness, the patient self-medicated with paracetamol (3–4 tablets/day), ibuprofen 400 mg (1–2 tablets/day), aspirin in antiplatelet doses (2 tablets/day); the patient refused to be medically evaluated during the illness;
- cardiovascular pathology was noted in the patient’s family history—essential arterial hypertension in both parents and polyglobulia (father);
- the patient’s personal pathological history was essential hypertension grade II (highest value of Blood Pressure 180/90 mmHg), controlled under monotherapy with bisoprolol 5 mg/day, frequent palpitations with the subjective perception of an irregular heart rhythm, tachycardia, visible apex shock in the left V intercostal space on the medio-clavicular line in orthostatism;
- the patient had been constantly practicing strength sports (powerlifting and bodybuilding) for the past 16 years. To improve his performance, the patient had used AASs in a continuous cycle since the age of 18. In the last 6 months before his death, the patient had used Sustanon 250 mg/mL (3 doses/week: Monday, Wednesday, Friday), Nandrolone decanoate 100 mg/mL (administration rate identical to Sustanon), Trenbolone acetate 100 mg/mL alternatively with Methenolon enanthate (2 doses/week). The patient also used, more or less regularly, fast-acting insulin, growth hormones and derivatives (GHRP-6 peptides, ipamorelin, vermotropin), multivitamins, omega 3-6-9 fatty acids (3–4 capsules daily), linseed oil, high molecular weight carbohydrate powder, isolated protein powder 2 g/body kg/day, creatine, caffeine-based energy drinks. In the last 2–3 years, the patient was doing 5 bodybuilding workouts/week, without cardio type workouts, and his diet consisted of 3 main meals with a total nutritional value of less than 3000 kcal.

**Figure 1:**
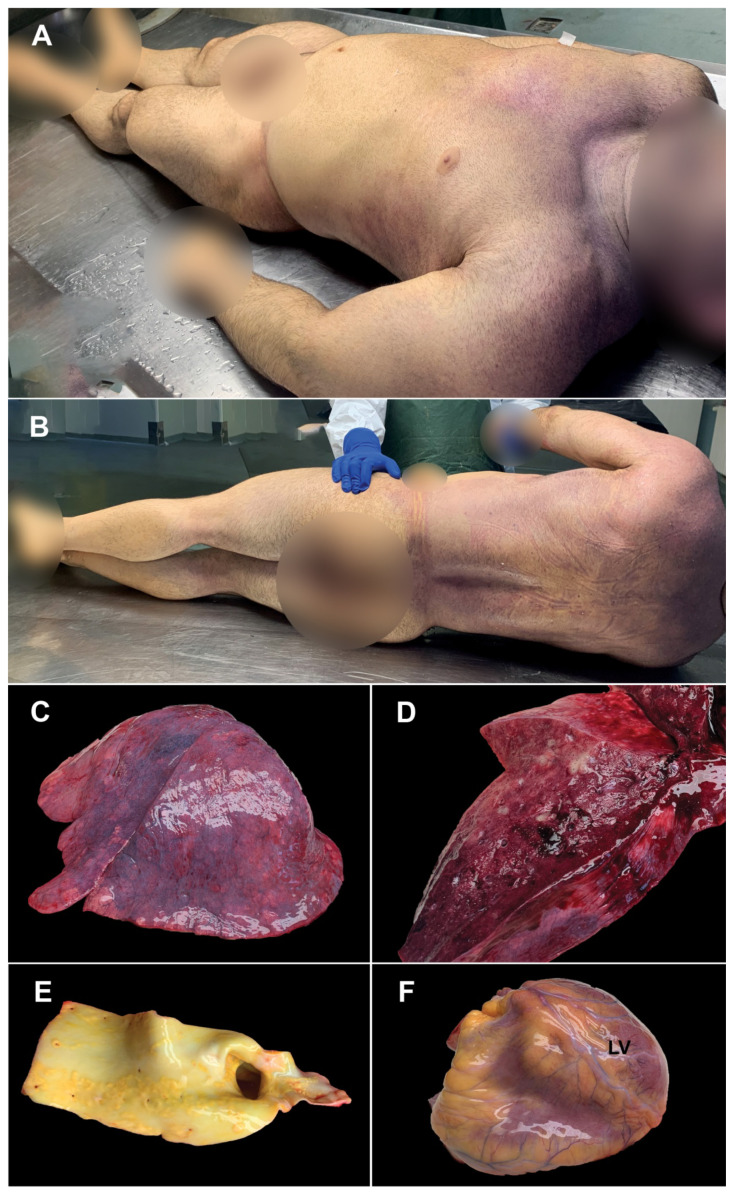

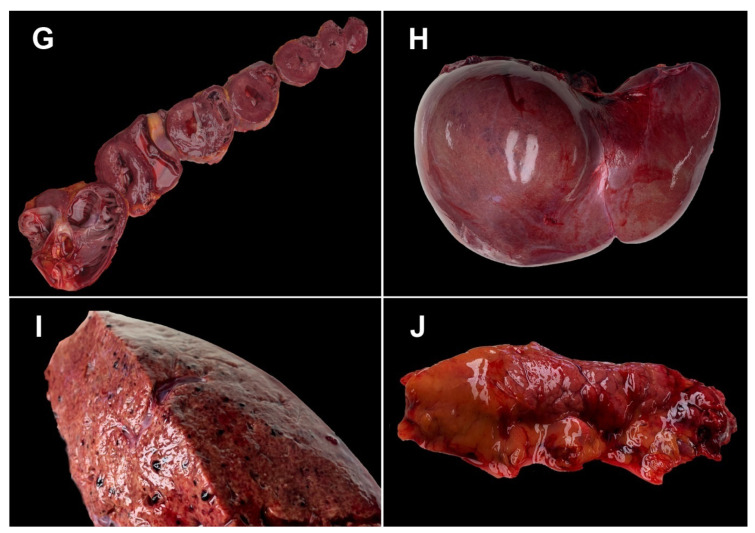
Macroscopic autopsy findings: (**A**,**B**)—Marked muscular hypertrophy; (**C**)—violet pulmonary surface, with multiple reddish hematic suffusions (microhemorrhages); (**D**)—pulmonary section, dark red blood, and foamy, pinkish fluid oozes. (**E**)—atherosclerosis of the descending aorta; (**F**)—heart hypertrophy (LV—left ventricle); (**G**)—transversal serial sections through the heart, with the highlighting of the old infarction; (**H**)—hepatic dystrophy; (**I**)—hepatic section; (**J**)—pancreas hypertrophy.

**Macroscopic autopsy findings.** Necropsy was performed 24 h postmortem. On external examination—male corpse, 165 cm tall, 85 kg, BMI 31.22, marked muscle hypertrophy (Figure 1A,B), no signs of putrefaction, with “cape” cyanosis (Figure 1A).

The lungs had increased weight (right lung 965 g, left lung 790 g), violet color; on the pleuro-pulmonary surface, bilaterally, multiple reddish hematic suffusions, with a diameter of 0.1–0.2 cm, could be detected (Figure 1C). When palpating the lungs, increased consistency was perceived, without crepitations. In the section, a compact, glossy appearance was observed; a significant amount of dark red blood and foamy, pinkish fluid oozes (Figure 1D).

The aorta showed numerous yellowish atherosclerotic plaques, raised on the surface of the intima (Figure 1E). The heart (460 g) showed myocardial hypertrophy (Figure 1F,G), the wall of the left ventricle had a maximum thickness of 3 cm, and the interventricular septum—2.5 cm. On the cross-section, a gray-whitish area of sclerosis (old myocardial infarction, Figure 1G) could be identified on the left ventricular wall. The liver had a significantly increased weight (2735 g) and was yellowish-brown in color (Figure 1H,I). The pancreas also showed significantly increased dimensions (weight 145 g) (Figure 1J).

**Figure 2:**
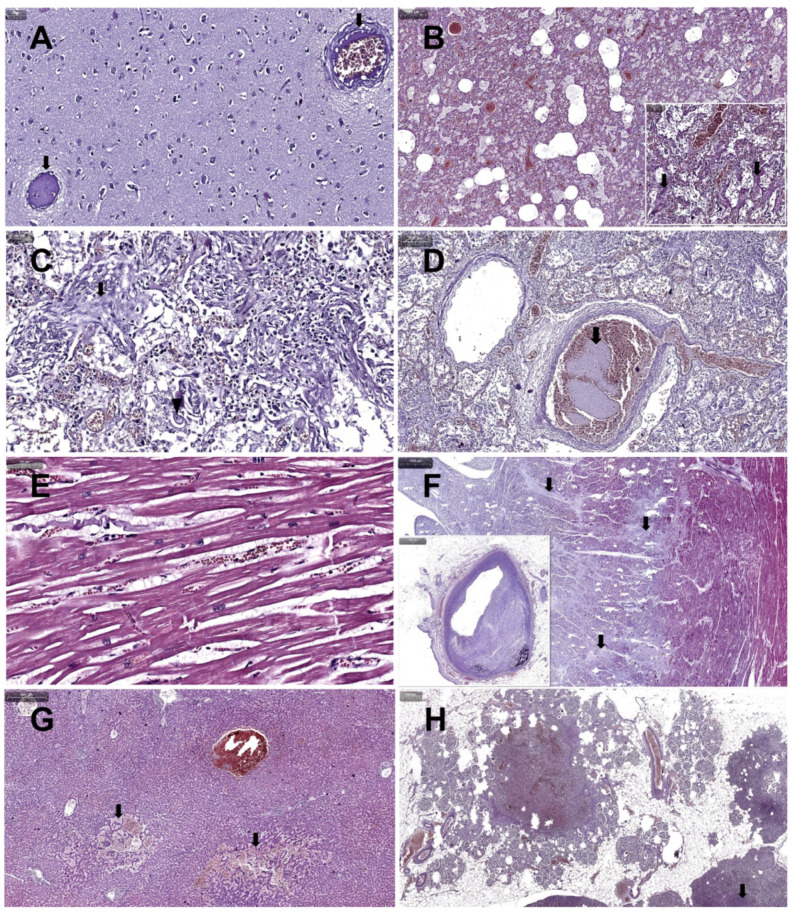
Results of the histopathological examination, HE: (**A**)—microthrombi with endothelitis (arrows) in cerebral tissue, on cerebral edema background; (**B**)—stasis, emphysema and massive pulmonary edema with DAD grade 1 (detail arrows); (**C**)—focal organizing pneumonia (arrow) and alveolar giant macrophages with nuclear viral inclusion (arrow head); (**D**)—pulmonary thromboembolism (arrow); (**E**)—hypertrophic myocadiac muscular fibers; (**F**)—old subendocardial myocardial infarction (arrows), associated with significant coronary artery occlusion by advanced atheromatous plaque (insert); (**G**)—hepatic peliosis (arrows); (**H**)—severe pancreatic nesidioblastosis and pancreatic multifocal necrosis (arrow).

The results of the histopathological examination showed microthrombi with endothelitis (arrows) in cerebral tissue, on a cerebral edema background (Figure 2A), in pulmonary tissue—stasis, emphysema and massive pulmonary edema (Figure 2B) with diffuse alveolar damage (DAD) grade 1 (Figure 2B detail arrows), focal organizing pneumonia (Figure 2C arrow) and alveolar giant macrophages with nuclear viral inclusion (Figure 2C arrow head), pulmonary thromboembolism (Figure 2D arrow). The heart showed hypertrophic myocadiac muscular fibers (Figure 2E), old subendocardial myocardial infarction (Figure 2F arrows), associated with significant coronary artery occlusion by advanced atheromatous plaque (Figure 2F insert). The liver presented hepatic peliosis (Figure 2G arrows), and the pancreas—severe nesidioblastosis and multifocal necrosis (Figure 2H).

Results of the immunohistochemical examinations are presented in the Table 1.

**Molecular biology.** The RNA test was positive for SARS-CoV-2, and the amplification of all three targets were observed: SARS-like coronaviruses gene (Ct = 25,274), E gene (Ct = 21,848) and N gene (Ct = 25,915). The test for identification of SARS-CoV-2 variant indicated the genetic line B.1.258.

The following HLA allele were identified: A*03, A*25, B*18, B*35, C*04, C*12, DRB1*04, DRB1*15, DQB1*03, DQB1*06.

## 4. Discussion

**Genetic data.** Data literature show that the HLA allele pairs identified by us in the studied patient correlate with severe forms of the disease (HLA-DRB1*04) [5], and prolonged duration of the disease (DQB1*03:02) [6]. In a group of 99 Italian patients affected by a severe or extremely severe form of COVID-19, HLA allele frequency distribution demonstrated a significant association for HLA-DRB1*15:01 and HLA-DQB1*06:02 compared to a reference group of 1017 Italian individuals [7]. One study gives a higher rate of the HLA-A*03 allele in COVID-19 patients than in healthy controls [8] and another study showed a positive log-linear correlation of A*25 allele with COVID-19 incidence rate [9]. Inspection of COVID-19 disease severity outcomes reveal significant risk associations with C*04:01 [10,11].

The genetic line identified in our case, B.1.258, had been circulated in Central Europe since August 2020, long before the import of B.1.1.7 [12].

**Testosterone and COVID-19.** Synthetic analogues of testosterone are the most widely used AASs [13].

Regarding the correlation between testosterone levels and severe forms of COVID-19, a prospective study of 358 COVID-19 patients described a correlation between low serum testosterone and a poor prognosis, finding that low testosterone was linked to more severe forms of COVID -19, a need for intensive care and death [14]. However, that study did not establish whether initially low testosterone predisposed patients to worse COVID -19-related outcomes, or whether the infection with SARS-CoV-2 caused lower testosterone levels, with more severe infections potentially leading to larger decreases in testosterone. Another study, on a smaller sample of 81 COVID-19 patients, identified the same connection between low testosterone levels and poorer COVID-19 outcomes, albeit without reaching statistical significance [15].

However, the link between androgen signaling and SARS-CoV-2 infectivity has been established and is believed to explain, at least in part, why men tend to have more severe forms of COVID-19 compared to women [16]. Although the determination of serum testosterone concentration was not performed in the case of our patient, its values were certainly above the physiological limit at the time of SARS-CoV-2 infection, negatively influencing the patient’s immune system [17,18,19].

Authors believe that the thromboembolic phenomena of this case (Figure 2A,D) are due to both AASs and a background of a genetic predisposition (father with polyglobulia) and to the COVID-19 infection. Testosterone causes erythrocytosis, a well-established side effect [20]. Although the mechanism linking thromboembolism to AASs abuse has not been elucidated, reviews on this topic present a broad consensus linking AASs abuse to an increased risk of developing thromboembolism, among other cardiovascular disorders [21,22]. Another review explains that, although AASs can also lead to enhanced fibrinolysis, their net overall effect remains procoagulant, maintaining consensus with the previously mentioned studies [23]. On the other hand, the risk of thromboembolism in COVID-19 is already well known [24,25].

**Testosterone and immunity.** Sexual dimorphism in immunity has been widely studied, and the consensus is that females have more active immune systems compared to males, meaning that they are better equipped to combat various infections, while at the same time being more susceptible to autoimmune diseases. This is a result of both hormonal and genetic factors, and their individual contributions could not be fully separated [26,27]. A review on the role of testosterone concluded that it has an overall immunosuppressive effect mediated by its action on a host of innate and adaptive immune cells [28].

The effect of AASs use on immunity has not been widely studied. A small study on 13 healthy bodybuilders using AASs showed marginal reductions in serum IgG, IgA and IgM levels compared to non-AASs users [29]. Some case studies appear to link AAS-related immunosuppression to increased disease severity in conditions such as severe rhinovirus pneumonia [30], septic shock with acute respiratory distress syndrome [31], necrotizing myofasciitis [32] and recurring herpes zoster ophthalmicus infection [33].

While the influence of testosterone or AASs on the immune response requires further study, they both have immunosuppressive effects that may reasonably lead to poorer outcomes when fighting infection. Thus, the case studied had a low immune status at the time of infection.

**Histopathological changes.** Among the cardiovascular disorders caused by AASs abuse, myocardial infarction is widely reported, and is most often triggered by atherosclerosis (Figure 1E and Figure 2F), increased thrombogenesis or vasospasms [34]. In most cases, peliosis hepatis (Figure 2G) has been associated with long-term AASs use; in the event of intraperitoneal hemorrhages, it can endanger the patient’s life [35].

Reports of nesidioblastosis (Figure 2H) in adults are extremely rare. The cause of nesidioblastosis in adults is unknown, but it could either be a genetic defect similar to those causing congenital hyperinsulinism [36]. In all reports, nesidioblastosis is linked to hyperinsulinism. No reports were found connecting nesidioblastosis to the use of exogenous insulin, AASs or growth hormone. Growth hormones encourage the growth of tissue (Figure 1H,J) and create a hyperglycemic environment in the body [37].

**Figure 3:**
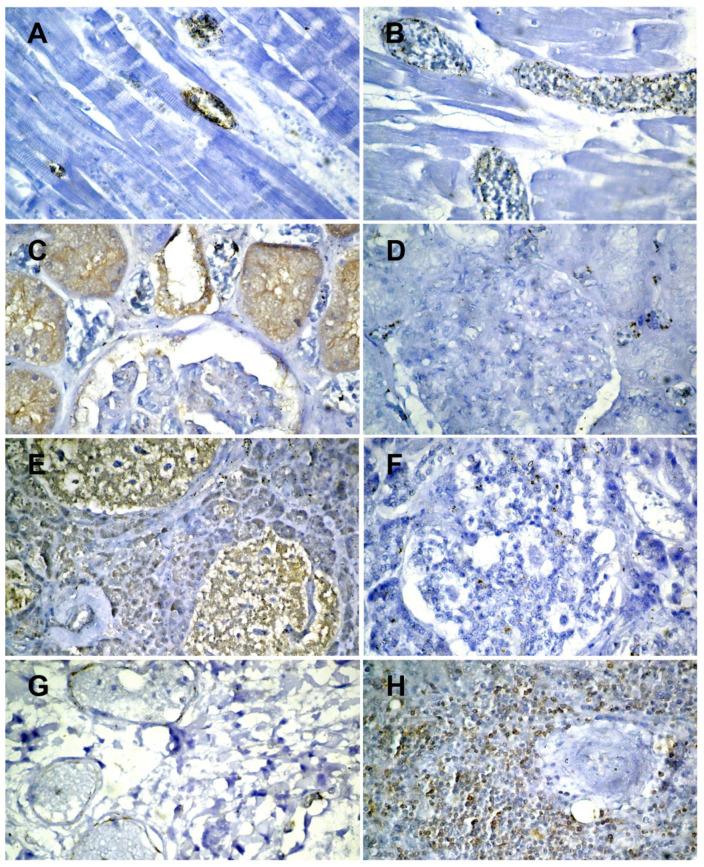

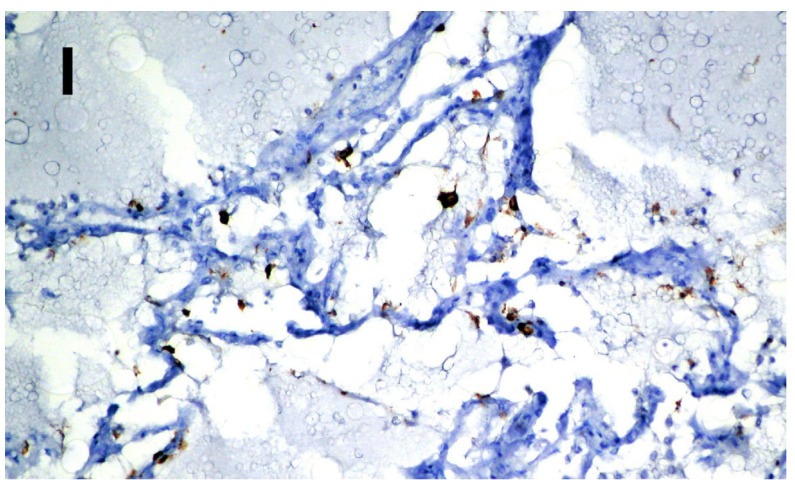
Results of the immunohistochemical examinations: (**A**)—ACE-2 focal positive on the endothelium of intramyocardial vessels (×400); (**B**)—SARS-CoV-2 focally positive on the endothelium of intramyocardial vessels (×400); (**C**)—ACE-2 intensely positive on the proximal tubules and corresponding arteriole of the juxtaglomerular apparatus (×400); (**D**)—SARS-CoV-2 focally positive on intertubular capillaries (×400); (**E**)—ACE-2 intensely positive on the beta cells of the islets of Langerhans (×200); (**F**)—SARS-CoV-2 focally positive on the beta cells of the islets of Langerhans (×400); (**G**)—ACE-2 positive on the endothelium of pulmonary vessels (×400); (**H**)—ACE-2 positive on lymphocytes from splenic follicles (×400); (**I**)—SARS-CoV-2 positive on alveolocytes (×400).

The pulmonary histopathological changes observed (Figure 2B–D) are already known and described in the COVID-19 literature [38,39,40]. The human pancreas can be a target of SARS-CoV-2 infection, and β-cell infection (Figure 3F) could contribute to the metabolic dysregulation/diabetes observed in patients with COVID-19 [41]. Studies on necropsic kidney fragments (immunostaining and in situ hybridization) suggest that SARS-CoV-2 is present in various segments of the nephron [42].

The lack of laboratory examinations and antemortem radio-imaging investigations, as well as the lack of serum determination of the postmortem AASs concentration, represent limitations to our study.

## 5. Conclusions

In the case analyzed, the severe form of COVID-19 was due to several factors: genetic predisposition, long-term abuse of AASs, which favored the development of a pluriorganic pathological tissue terrain, and recent consumption of AASs, which influenced the immune system at the time of infection. The main reason of death was COVID-19. Severe lung damage was followed by multiple organ failure and death.

## Figures and Tables

**Table 1:** Results of the immunohistochemical examinations.

	ACE 2	SARS-CoV-2
Brain	Negative	Negative
Myocardium	Positive on the vascular endothelium (Figure 3A)	Positive on the vascular endothelium (Figure 3B)
Lung	Positive on the vascular endothelium (Figure 3G)	Positive on alveolocytes (Figure 3I)
Spleen	Positive on the lymphocytes in the follicles (Figure 3H)	Negative
Kidneys	Positive on proximal tubules and on the endothelium of the corresponding arteriole (Figure 3C)	Positive on intertubular capillaries (Figure 3D)
Liver	Positive on the biliary epithelium, the endothelium of the centrilobular venules	Negative
Pancreas	Positive on the islets of Langerhans (beta cells) (Figure 3E)	Positive on the islets of Langerhans (beta cells) (Figure 3F)

## Data Availability

The autopsy results are available at the Institute of Legal Medicine in Cluj-Napoca Romania; Contact: cvsiserman@gmail.com. The immunohistochemistry results are available at the Department of Anatomy and Embryology, Iuliu Hatieganu University of Medicine and Pharmacy, Cluj-Napoca, Romania; Contact: jeican.ionut@umfcluj.ro. The genetic data and molecular biology results are available at the Department of Cell and Molecular Biology, Iuliu Hatieganu University of Medicine and Pharmacy, Cluj-Napoca, Romania; mvica@umfcluj.ro; The virology analysis results are available at the Viral Respiratory Infections Laboratory, Cantacuzino National Military-Medical Institute for Research and Development, Bucharest, Romania; Contact: lazar.mihaela@cantacuzino.ro.
